# Combination of 1.1 mm flexible cryoprobe with conventional guide sheath and therapeutic bronchoscope in biopsy of apical upper lobe solitary pulmonary nodule

**DOI:** 10.1186/s12890-020-01199-3

**Published:** 2020-06-03

**Authors:** Sze Shyang Kho, Chan Sin Chai, Larry Ellee Nyanti, Adam Malik bin Ismail, Siew Teck Tie

**Affiliations:** 1 Division of Respiratory Medicine, Department of Medicine, Sarawak General Hospital, Ministry of Health, Kuching, Sarawak Malaysia; 2 Department of Pathology, Sarawak General Hospital, Ministry of Health, Kuching, Sarawak Malaysia

**Keywords:** Cryobiopsy, Case report, 1.1 mm cryoprobe, rEBUS, Solitary pulmonary nodule

## Abstract

**Background:**

Lung cancer is frequently situated peripherally in the upper lobes of the lung. Acquiring adequate tissue from this difficult-to-reach area remains a challenge. Transbronchial cryobiopsy (TBCB) has the ability to acquire larger specimens, but the rigidity of the standard 1.9 mm and 2.4 mm cryoprobes frequently poses challenges when used with a guide sheath (GS). The novel 1.1 mm cryoprobe, being both smaller and more flexible, may address this limitation. We describe the usage of this 1.1 mm flexible cryoprobe with GS in the biopsy of solitary pulmonary nodules (SPN) in the apical segment of the upper lobe in two cases.

**Case report:**

Both procedures were conducted with advanced airway under total intravenous anaesthesia. 2.6 mm GS was used in combination with a 2.2 mm rEBUS probe, using a therapeutic bronchoscope. Case 1 describes a SPN in the apical segment of the right upper lobe that was inconclusive by forceps biopsy due to GS displacement and inadequate biopsy depth. A steerable GS combined with the novel cryoprobe subsequently overcame this issue. Case 2 describes a SPN in the apical segment of the left upper lobe in which the standard cryoprobe failed to advance through the GS due to steep angulation. It also highlights with shorter activation time, the novel cryoprobe enable biopsied tissue to be retrieved through the GS while the bronchoscope-GS remains wedgend in the airway segment. There were no bleeding or pneumothorax complications in both cases, and histopathological examination confirmed adenocarcinoma of the lung.

**Conclusion:**

The 1.1 mm flexible cryoprobe in combination with GS and therapeutic bronchoscope offers an option to acquire adequate tissue in difficult-to-reach regions in the lung such as the apical segment of upper lobes. Further prospective series to evaluate its performance and safety in SPN biopsy is highly anticipated.

## Background

Lung cancer has a predilection for the upper lobes (64.1%) and peripheral regions (72.7%) [1]. Biopsy of upper lobe solitary pulmonary nodules (SPN) via guided bronchoscopy is both technically challenging owing to the large amount of required angulation, and may carry a higher risk of procedural complications [2].

The conventional guide sheath (GS) is an adjunctive tool which acts as an extended bronchoscopic conduit by facilitating the placement of forceps or radial endobronchial ultrasound (rEBUS) probes beyond the maximal distal bronchi reachable by bronchoscope. GS placement combined with rEBUS verification has been shown to increase the diagnostic yield of SPN by ensuring a reproducible biopsy site [3]. Cryobiopsy provides larger specimens compared to conventional forceps and has been demonstrated to increase the diagnostic yield of SPN [4]. However, the stiffness of the standard 1.9 mm and 2.4 mm cryoprobes make it difficult to be inserted into the GS, especially into apical segments of the upper lobe [5, 6]. A novel, smaller, commercially available 1.1 mm flexible cryoprobe may address this limitation.

We hereby describe two cases of SPN located in the apical segment of upper lobes which were successfully biopsied by the novel 1150 mm 1.1 mm flexible cryoprobe in combination with conventional GS.

## Case presentation

### Case 1

A 74 year old lady, non-smoker, presented with an incidental finding of elevated serum carcinoembryonic antigen of 177.4 ng/ml during a routine medical checkup. ^18^F-FDG PET/CT revealed a dominant hypermetabolic nodule at the apical segment of right upper lobe (RUL), along with multiple ipsilateral lung nodules and bone metastasis. The target lesion measured 2.23 cm in diameter, demonstrated positive CT bronchus sign and was mapped to RB1aii (posterior-medial sub-segment of the RUL apical segment). Written consent was obtained from the patient for rEBUS guided bronchoscopic biopsy.

Under conscious sedation, flexible bronchoscopy (*BF-1TH190, Olympus Medical, Japan*) was performed trans-nasally under fluoroscopic guidance. Initial airway inspection revealed normal airway. A conventional 2.6 mm GS (*SG-201C, Olympus Medical, Japan*) was loaded with a bidirectional guiding device (*CC-6DR-1, Olympus Medical, Japan*) to assist its placement into RB1aii. Due to the extreme medial location of the target lesion, multiple intra-procedural maneuvering was required to achieve accurate placement. A 2.2 mm 20 MHz rEBUS (*UM-S20-20R, Olympus Medical, Japan*) probe inserted via the GS confirmed an eccentric orientated lesion subsequently. Biopsy was then performed using a standard 2.0 mm biopsy forcep (*FB-231D, Olympus Medical, Japan*) under fluoroscopic guidance. However, significant lateral displacement of GS was observed each time the forcep was inserted. Despite performing a total of 10 biopsies, histological examination only revealed bronchial epithelium. As the patient was not under advanced airway, cryobiopsy was not performed in that setting and the patient consented for a repeat procedure with cryobiopsy.

**Fig. 1:**
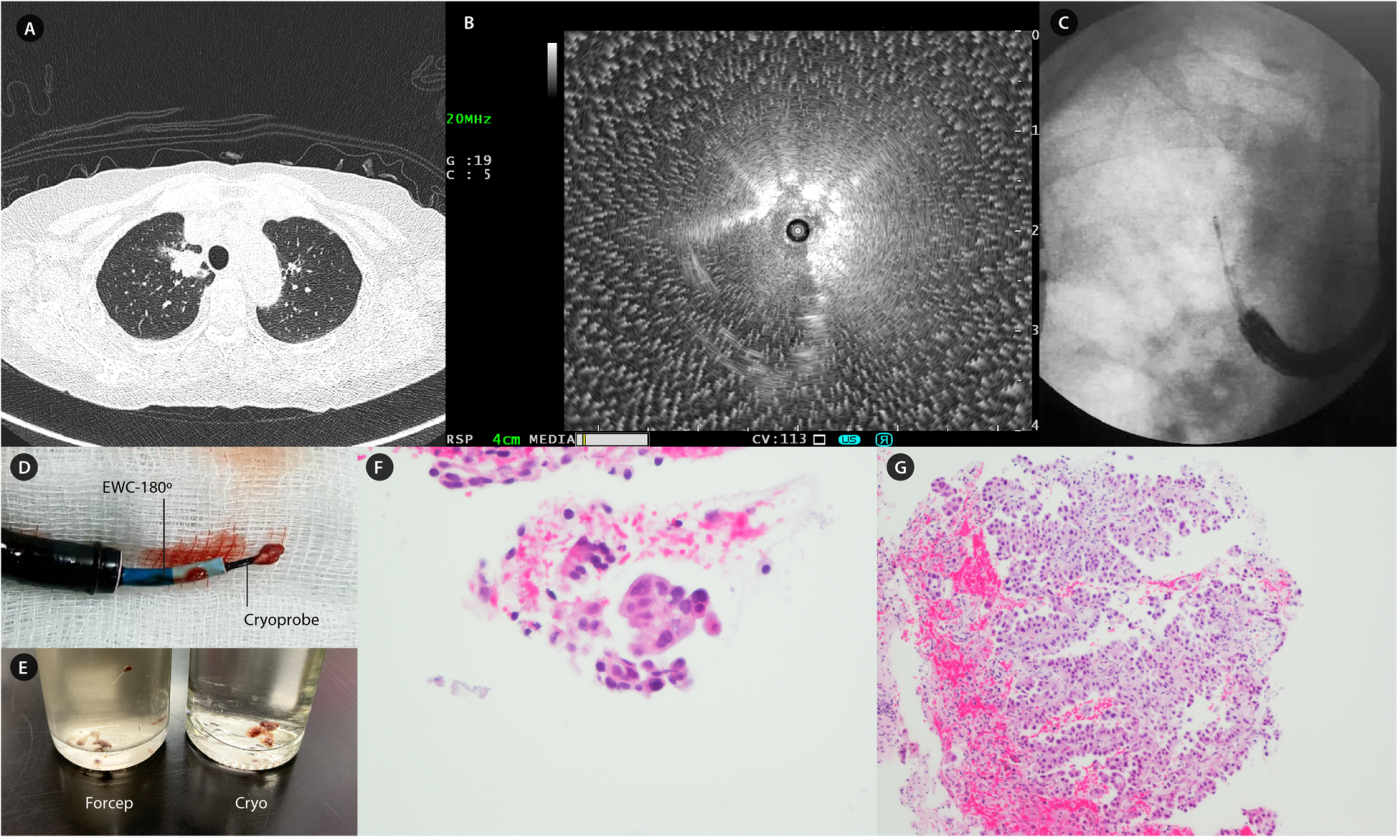
Case 1 – Solitary pulmonary nodule in apical segment of right upper lobe. **a**: 2.23 cm solitary pulmonary nodule in apical segment (medial sub-segment) of right upper lobe. **b**: An eccentric rEBUS orientated lesion localized at RB1aii with no surrounding vessel. **c**: Placement of 1.1 mm flexible cryoprobe through GS into the target lesion at apical segment of right upper lobe under fluoroscopic guidance. **d**: Retrieved cryobiopsy specimen upon *en bloc* removal of bronchoscope-GS-cryoprobe. **e**: Gross appearance of fragmented forceps biopsy in comparison with cryobiopsy specimen. **f**: forceps biopsy revealed fragmented bronchial tissue with rare cluster of atypical cells (*Hematoxylin & Eosin, × 400 magnification*). **g**: Cryobiopsy revealed fragments of alveolated parenchymal infiltrated with tumour cells in glandular pattern, exhibiting enlarged and pleomorphic nuclei (*Hematoxylin & Eosin, × 100 magnification*). Immunohistochemistry shows diffuse and strong positivity to TTF-1, consistent with adenocarcinoma of lung

The patient was induced under total intravenous anaesthesia (TIVA) with intravenous proprofol and remifentanil and intubated with an 8 mm endotracheal tube. A steerable 1070 mm, 2.6 mm 180^o^ extended working channel catheter (EWC*, Edge™ Firm Tip, Medtronic, Minneapolis*) was used as the GS this time due to anticipated acute angulation. rEBUS confirmed the GS location, showing an eccentric orientated lesion with no significant surrounding vessels. Similarly, initial biopsy attempts with conventional forcep was met with significant deflection of the GS as the forcep passed through the final curvature. We then deployed the conventional 1.9 mm cryoprobe (*ERBE, Medizintechnik, Tϋbingen, Germany*), but it encountered tremendous resistance at the bronchoscope curvature point. Finally, a decision was made to utilize the novel 1.1 mm flexible cryoprobe, which was inserted effortlessly via the GS into the target lesion under fluoroscopic guidance without any GS displacement. The cryoprobe was first activated for 3 s, after which the bronchoscope-GS-cryoprobe unit was removed *en bloc*. The target was then re-navigated and cryobiopsy was successfully performed twice more with 5- and 7-s activation each. Minimal post biopsy bleeding was observed intra-procedure and no pneumothorax occurred. On histopathological examination and immunohistochemistry analysis, forceps biopsy only yielded fragmented bronchial epithelium with rare atypical cells while cryobiopsy yielded adenocarcinoma lung with sensitizing epidermal growth factor receptor (EGFR) mutation. Figure 1 illustrates the essence of this case.

### Case 2

A 66 year old lady, non-smoker, presented with prolonged cough with loss of appetite. Chest radiograph noted a left upper lobe (LUL) nodule. Tuberculosis workup was negative and CT thorax confirmed a 2.7 cm SPN with positive CT bronchus sign at the apico-posterior segment of the LUL with no suspicious mediastinal and hilar lymph node or distant metastasis. The target lesion was mapped to LB1 + 2ai (lateral-superior sub-segment of the LUL apico-posterior segment). Patient consented for rEBUS guided bronchoscopic transbronchial cryobiopsy as the CT bronchus sign was truncated at the edge of lesion which may preclude a successful forceps biopsy.

**Fig. 2:**
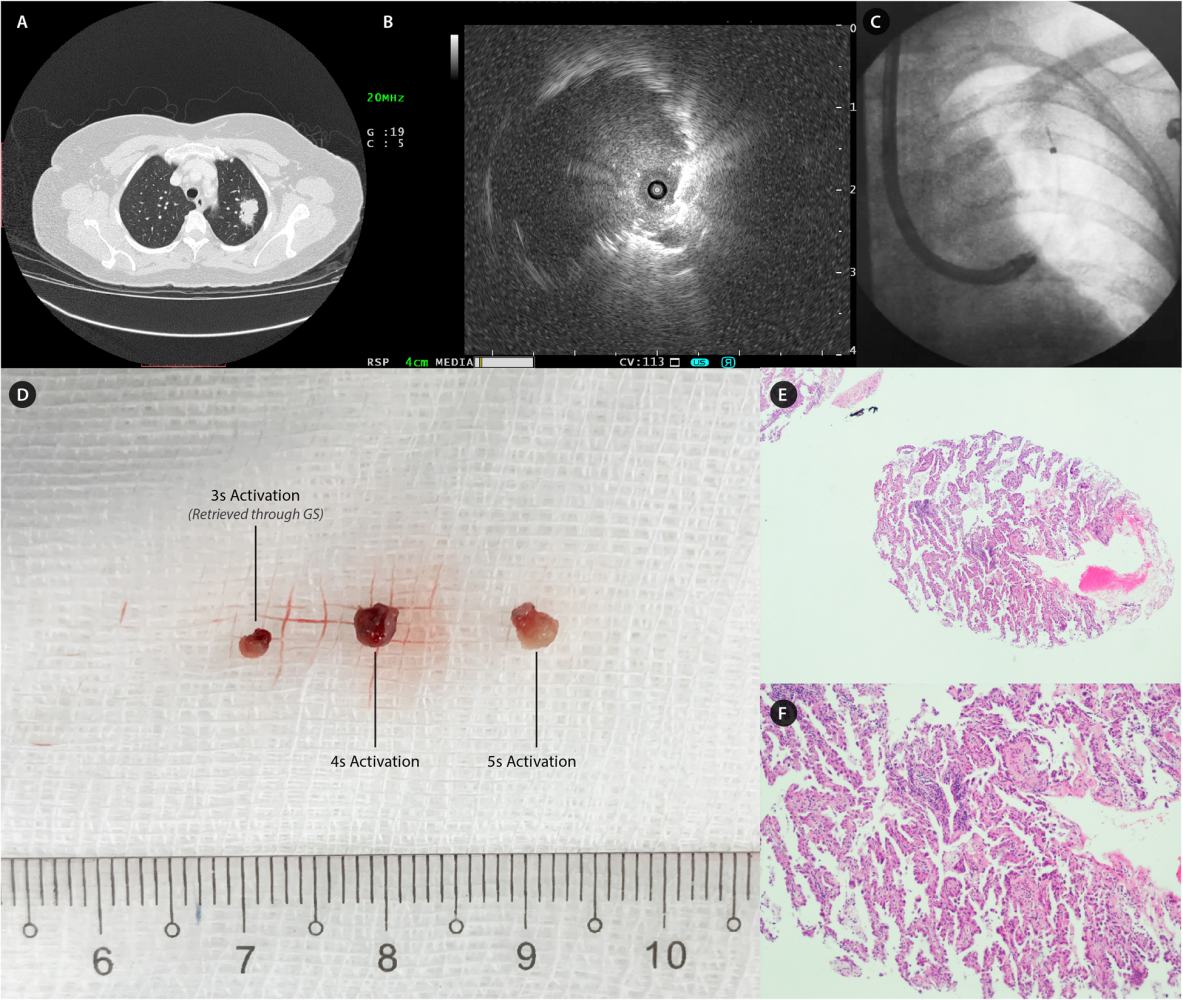
Case 2 – Solitary pulmonary nodule in apical segment of left upper lobe. **a**: 2.7 cm solitary pulmonary nodule in apical segment of left upper lobe. **b**: An eccentric rEBUS orientated lesion localized at LB1 + 2ai with no surrounding vessels. **c**: Placement of 1.1 mm flexible cryoprobe through GS into the target lesion at apical segment of left upper lobe under fluoroscopic guidance. **d**: Gross appearance of cryobiopsy specimen (left to right): 2 mm with 3 s activation (*specimen retrieved through GS*), 3 mm with 4 s activation and 3 mm with 5 s activation. **e**: Structural integrity of lung tissue was maintained with cryobiopsy which revealed fibrotic alveolar tissue infiltrated by tumour. (*Hematoxylin & Eosin, × 100 magnification*). **f**: High power examination revealed tumour infiltration arranged in glandular pattern with large and round pleomorpic hyperchromatic nuclei and prominent nuceloli. Immunohistochemistry was positive for CK7 and TTF-1, consistent with adenocarcinoma of lung. (*Hematoxylin & Eosin, × 400 magnification*)

The patient was intubated with an 8 mm endotracheal tube under TIVA. Airway examination using a therapeutic bronchoscope revealed normal airways. An eccentric lesion was localized to LB1 + 2ai by rEBUS with a conventional GS (*SG-201C, Olympus Medical, Japan*). No adjacent vessel was noted on rEBUS and the nearest vessel measured 1.8 mm on CT scan. Under fluoroscopic guidance, 10 forcep biopsies were performed via GS uneventfully. After re-verification by rEBUS/GS, the 1.9 mm cryoprobe was then inserted into the GS but failed to advance past the bronchoscope curvature point due to tremendous resistant. The novel 1.1 mm cryoprobe was then inserted into the GS without difficulty and the tip was then smoothly maneuvered into the site of interest under fluoroscopic guidance. Cryobiopsy was activated for 3 s and the probe was removed swiftly from the GS leaving the bronchoscope and GS in place. Tissue was thawed in normal saline and fixed in formalin solution immediately. Minimal bleeding was observed from LB1 + 2 ostium as the GS remained wedged for tamponade effect. The cryoprobe was then re-inserted into the GS and placed into the target lesion under fluoroscopic guidance. For the second attempt, cryobiopsy was activated for 4 s but the probe was unable to be retrieved through the GS and the bronchoscope-GS-cryoprobe had to be removed *en-bloc* to retrieve the tissue. The last attempt was performed after re-navigation; cryoprobe was activated for 5 s and all instruments were then removed *en-bloc*. Minimal bleeding was encountered intra-procedure and no pneumothorax occured. Macroscopically, the cryobiopsy specimen measured 2-3 mm in diameter. Histopathological examination with immunohistochemistry analysis revealed invasive adenocarcinoma of the lung with sensitizing epidermal growth factor receptor (EGFR) mutation from both forcep and cryobiopsy specimens. This case was illustrated in Fig. 2.

## Discussion

Optimal tissue acquisition is crucial in the age of targeted therapy and immunotherapy for lung cancer, hence it is paramount that biopsy tools are able to acquire large tissue samples while being able to access difficult-to-reach areas of the lung. Transbronchial cryobiopsy (TBCB) is widely used in the diagnosis of diffuse parenchymal lung disease (DPLD), the use of which has been standardized through expert recommendations [7]. However, its role in SPN diagnosis remains uncertain although various studies have demonstrated its feasibility, especially if lesions are eccentric or adjacently orientated to rEBUS [4, 8]. However, the conventional cryoprobe is stiff and may not be easily inserted into all regions of the lung, especially in highly angulated bronchi such as the apical segment of the upper and lower lobe [5]. In contrast, the novel commercially available 1150 mm 1.1 mm flexible cryoprobe offers increased flexibility. Animal studies on the early prototype model showed promising results in terms of feasibility and superior specimen quality when biopsy was performed in bilateral upper and lower lobes [9, 10].

One of the strengths of the novel 1.1 mm cryoprobe is its small size, thus allowing easy insertion into any conventional GS and the ability to navigate through the steep curvature leading into the apical segment of the upper lobes as demonstrated in both our cases. Although the conventional cryoprobe can be inserted into the target segment without GS, the actual biopsy site may not be accurately reproducible due to the shape memory property of the cryoprobe. It is also an established fact that GS is an important tool to ensure successful SPN biopsy [5, 6]. Another advantage of the novel 1.1 mm cryoprobe lies with its excellent flexibility. The novel 1.1 mm cryoprobe did not produce significant GS deflection during insertion, thus ensuring a consistent biopsy site after rEBUS verification. Both types of commonly used GS (*SG201C and EWC180*^*o*^) were easily deflected even by standard biopsy forcep as demonstrated in Case 1, which translated to ineffective biopsy. Future improvements in GS design to allow more maneuverability while maintaining longitudinal stability may address this dilemma. Utilization of a thin caliber bronchoscope which itself acts as a GS may be a potential companion to the novel 1.1 mm cryoprobe [11]. However, although thin caliber bronchoscope may provide better access, specimen retrieval via a smaller working channel would possess a great challenge. Hence, the potential benefit of having instruments remain wedged after cryobiopsy is less likely to happen.

While the commercial 1.1 mm cryoprobe comes equipped with a 817 mm 2.6 mm over-sheath, this is too short to be used as a GS for SPN biopsy, as it was originally designed to allow the bronchoscope to remain in the proximal segmental airway during specimen retrieval in transbronchial cryobiopsy for DPLD [12]. In Case 2, we demonstrated that an activation of 3 s allowed tissue retrieval via the longer 1150 mm conventional GS while the bronchoscope remained wedged, but not if activation was more than 3 s. However, the optimal activation time to allow tissue retrieval via the longer conventional GS remains unknown. Early prototype studies using the shorter 817 mm oversheath demonstrated possible tissue retrieval with activation up to 6–10 s [9, 10]. However, some challenges were described as no tissue was obtained in 2 cases with 4 s activation and 1 case demonstrated failure to retract the probe through the oversheath with 6 s activation [10].

Despite being relatively small in size, the 1.1 mm cryoprobe provides superior specimen quality when compared to standard biopsy forceps. In the prototype model, specimens retrieved from oversheath had a larger mean specimen size of 3.87 ± 2.89 mm^2^ with less crush effect compared to those obtained via 2.0 mm forcep [9]. Meanwhile, the commercially available model showed a mean specimen area of 4.7 ± 2.6 mm^2^ when retrieved from oversheath with 3–11 s activation and 13.1 ± 6.5 mm^2^ without oversheath when activated for 11–13 s in animal studies [13]. Crucially, it also demonstrated greater biopsy depth by providing more alveolar tissue than a standard 2.0 mm forcep [9, 10]. Biopsy depth is important in the sampling eccentric and adjacently orientated rEBUS lesions where mucosa invasion may not be complete. This was demonstrated in Case 1 in which forceps biopsy only yielded superficial biopsies repetitively. More studies to evaluate the optimum number of passes in transbronchial cryobiopsy of SPN using this novel 1.1 mm cryoprobe with or without GS are required.

There were a few challenges with the novel 1.1 mm cryoprobe. First, the optimal activation duration required for the longer GS and bronchoscope to remain in the airway during tissue retrieval has yet to be ascertained. As such, the blind period between bronchoscope withdrawal and reinsertion during cryobiopsy remains problematic, necessitating advanced airway for quicker re-entry access and prophylactic balloon blocker in the event of bleeding. Although usage of prophylactic balloon blocker is recommended for transbronchial cryobiopsy for DPLD, its placement in the upper lobe is usually challenging. Reassuringly, usage of rEBUS guided transbronchial cryobiopsy may potentially reduce bleeding complications by ruling out adjacent vessels at the biopsy site [14]. However, until further studies to assess the bleeding risk of this novel cryoprobe in combination with rEBUS/GS are available, transbronchial cryobiopsy for SPN should only be performed if the benefits outweigh the bleeding risk. Second, as the probe is significantly smaller than the conventional GS (2.6 mm), *en-bloc* removal with the bronchoscope and GS needs to be performed cautiously, with the assistant maintaining a firm grip on the cryoprobe at the GS ostium while the bronchoscopist holds the GS-Cryoprobe firmly at the bronchoscope working channel inlet to allow swift removal of bronchoscope-GS-cryoprobe as a single unit. Third, the single use design of the probe may be cost-prohibitive.

In conclusion, the 1.1 mm flexible cryoprobe is a promising tool to acquire adequate tissue in regions that are difficult-to-reach in the lung such as the apical segment of upper lobes. Further prospective series to evaluate its performance and safety in SPN biopsy is highly anticipated.

## Data Availability

Not applicable.

## Abbreviations

CT: Computed tomography
DPLD: Diffuse parenchymal lung disease
EGFR: Epidermal growth factor receptor
EWC: Extended working channel
^18^F-FDG PET/CT: ^18^F-labelled Fluoro-2-Deoxyglucose Positron Emission Tomography/CT
GS: Guide sheath
LUL: Left upper lobe
RUL: Right upper lobe
rEBUS: Radial endobronchial ultrasound
SPN: Solitary pulmonary nodule
TIVA: Total intravenous anaesthesia
